# Towards the optimisation of ceramic-based microbial fuel cells: A three-factor three-level response surface analysis design

**DOI:** 10.1016/j.bej.2019.01.015

**Published:** 2019-04-15

**Authors:** M.J Salar-García, A. de Ramón-Fernández, V.M. Ortiz-Martínez, D. Ruiz-Fernández, I. Ieropoulos

**Affiliations:** a Bristol BioEnergy Centre, Bristol Robotic Laboratory, Block T, UWE, Bristol, Coldharbour Lane, Bristol BS16 1QY, United Kingdom; b Department of Computer Technology, University of Alicante, Ctra. San Vicente del Raspeig s/n, 03690 Alicante, Spain; c Department of Chemical Engineering, University of Murcia, Campus de Espinardo s/n, 30100 Murcia, Spain

**Keywords:** 00-01, 99-00, Microbial fuel cells, Modelling, Response Surface Methodology, Ceramic membranes, Bioenergy

## Abstract

Microbial fuel cells (MFCs) are an environment-friendly technology, which addresses two of the most important environmental issues worldwide: fossil fuel depletion and water scarcity. Modelling is a useful tool that allows us to understand the behaviour of MFCs and predict their performance, yet the number of MFC models that could accurately inform a scale-up process, is low. In this work, a three-factor three-level Box–Behnken design is used to evaluate the influence of different operating parameters on the performance of air-breathing ceramic-based MFCs fed with human urine. The statistical analysis of the 45 tests run shows that both anode area and external resistance have more influence on the power output than membrane thickness, in the range studied. The theoretical optimal conditions were found at a membrane thickness of 1.55 mm, an external resistance of 895.59 Ω and an anode area of 165.72 cm^2^, corresponding to a maximum absolute power generation of 467.63 μW. The accuracy of the second order model obtained is 88.6%. Thus, the three-factor three-level Box–Behnken-based model designed is an effective tool which provides key information for the optimisation of the energy harvesting from MFC technology and saves time in terms of experimental work.

## Highlights

• Ceramic-based MFCs fed with urine for continuous bioenergy production.
• Response surface methodology for designing a sequential set of MFC experiments.
• Obtaining a second order model with a regression coefficient of 0.886.
• High effect of the external loading and anode area on the MFC power output.
• Membrane thickness does not show statistical influence on the MFC performance.

## 1 Introduction

Global warming along with depletion of fossil fuels are two of the most serious environmental issues for humankind. Microbial fuel cell (MFC) technology deals with both concerns from two different perspectives: (i) bioenergy production and (ii) wastewater treatment. MFCs are devices that benefit from microbial metabolism to turn the chemical energy stored in different kinds of organic substrates into electricity [1], [2], [3].

An MFC consists of an anodic chamber where bacteria oxidise the organic matter contained in a specific substrate producing electrons, protons, low amount of carbon dioxide and smaller molecules. Protons cross a selective separator to the cathode where combined with electrons, which come from the anode through an external circuit, complete the oxygen reduction reaction and form water. The anodic chamber hosts the anode electrode, usually made of porous carbonaceous materials due to their low cost and high bio-compatibility, which favour the biofilm growth. Regarding the cathode, carbon-based supports coated with a catalyst are commonly used. The redox reactions are completed by the reduction of an oxidant on the cathode, generally oxygen due to its abundance and high reduction potential. Noble metals, such as platinum, are commonly used for the oxygen reduction, however in recent years alternative low cost platinum-free materials have also been investigated to catalyse the oxygen reduction on the cathode (e.g. MnO_2_, iron-based materials, active carbon, etc.) [3], [4], [5].

Anodic and cathodic chambers are physically separated by a separator or selective membrane. The main functions for this separator are: (i) to maintain the separation between the electrodes, avoiding the short-circuit, (ii) to reduce substrate cross-over, protecting the cathode from fouling caused by both biological and inorganic compounds contained in the anodic chamber and (iii) to maintain the anaerobic condition in the anodic chamber, avoiding the oxygen transfer from the cathode to the anode. Commercial polymer-based membranes have commonly been used as separator in MFCs (e.g. Nafion, Ultrex, etc.), however their high cost limits the large-scale application of this technology. In recent years, alternative low cost materials have been investigated as MFC separators, being ceramic-based materials one of the most promising due to their low cost and their natural availability [6], [7].

Despite individual MFCs is still at an early stage of development, in the last few years scientific community focuses on demonstrating the implementation of this technology into practical applications [8], [9], [10]. In 2008, MFCs were successfully employed to power a meteorological buoy [11]. On the other hand, a recent research work reports that a similar MFC set-up to that used in the present work, also fed with urine, is able to power conventional electronic devices such as mobile phones. After 24 h, MFCs allow to charge up to 3.7 V the battery of the phone [12]. In addition to these promising results, the low cost of the materials employed open up the potential use of MFCs on telecommunication field in developing countries or remote locations. More recently, in 2017 Walter et al. reported some improvements regarding the use of ceramic-MFCs fed with urine to power different types of mobile phones. Authors concluded that a mobile phone charged by a MFC during 6 h is able to work over 3 h, including calls [13]. These results support the potential application of this technology as power supply for telecommunication purposes. However, in order to open up even more the range of real applications, it is necessary to optimise the energy harvesting from MFCs.

Modelling is a useful tool for analysing and optimising the behaviour of any system. These techniques allow us to save time and money since they cover multiple scenarios reducing the number of tests required. For these reasons, in the last few years the interest in modelling the behaviour of bioelectrochemical systems has increased significantly [14], [15]. Some of the computational models reported to optimise the performance of MFCs are focusing on the anode as the limiting factor. They consider that the biofilm growth is the key factor for a good-performing MFC. This category includes the one-dimensional model reported by Marcus et al. [16], which has subsequently served as the basis for other models focussing on the biofilm region. The authors describe the electron production from the substrate oxidation by using Monod and Nernst equations. Subsequently, Merkey and Chopp [17] developed a two-dimensional version of Marcus’ model which, apart from the biofilm region, also includes the bulk liquid, the solid electrode and their corresponding interfaces.

On the other hand, there are a few models which consider the overall cell including both anode and cathode chamber. In 2010, Zeng et al. [18] analysed the electrochemical performance of a double chamber MFCs by using Monod and Bulter-Volmer equations. Three years later, Oliveira et al. [19] included to this model the heat transport phenomena.

Finally, alternative models have been reported focused on modelling specific processes or elements of the system. For instance, Harnisch et al. [20] investigated the polarisation process around a Nafion-type membrane used as separator in a double chamber MFC. By contrast, Wen et al. [21] designed a model focuses on the polarisation and power curves of an air-cathode single chamber MFC.

As can be observed, significant efforts have been made in order to model and optimise MFCs. However, so far most of the models are based on MFCs fed with pure substrates or synthetic wastewater. Due to the complexity of the system, hardly ever MFCs using real wastes as fuel have been modelled [14], [15].

**Fig. 1:**
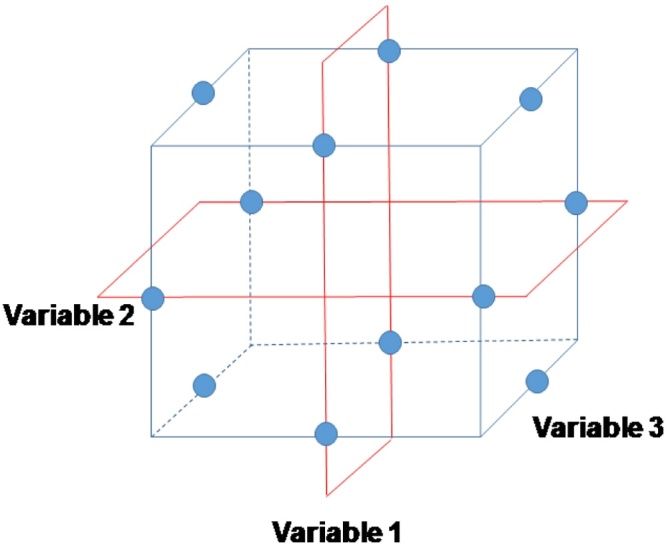
Cubical depiction of the Box–Behnken design.

Optimising the parameters involved in the MFC setup might reduce the cost of the technology and simultaneously maximise the energy harvesting. Statistical optimisation techniques allow us to search within a wide experimental domain with a minimum number of runs, saving money and time. Response surface methodology (RSM) is a statistical technique, which allows us to design a sequential set of experiments in order to achieve the optimal response. This method establishes a relationship between the input and output factors for the optimisation of the process. To this group belong central composite designs (CCD) or Box–Behnken designs (BBD). Whereas CCD is very similar to a factorial design but including different central points along the extreme value range, which improve the accuracy of the model in comparison with factorial design, BBD is not based on a factorial design [22], [23]. In this case, the experimental levels are located at the midpoints of the extreme value selected (see Fig. 1).

The main advantage of BBD is its efficiency because this design commonly requires fewer runs and they show rotatability or near-rotatability, which are desirable statistical properties. On the other hand, unlike both factorial design and CCD, BBDs do not test the corner point (extreme values) of the hypercube design which favour those experiments where perform trials in those regions are not feasible. BBD is a second-order model which allows us to determine a response surface of a specific system without a deep knowledge of its functioning as well as to maximise or minimise this surface in order to optimise the system [24], [25].

In this work, a three-factor three-level Box–Behnken design is used for the optimisation of ceramic-based MFCs fed with human urine. Key factors such as anode area, membrane thickness along with external resistance are investigated in order to evaluate their influence on the power output as well as maximise the MFC performance.

## 2 Material and methods

### 2.1 MFC configuration

**Fig. 2:**
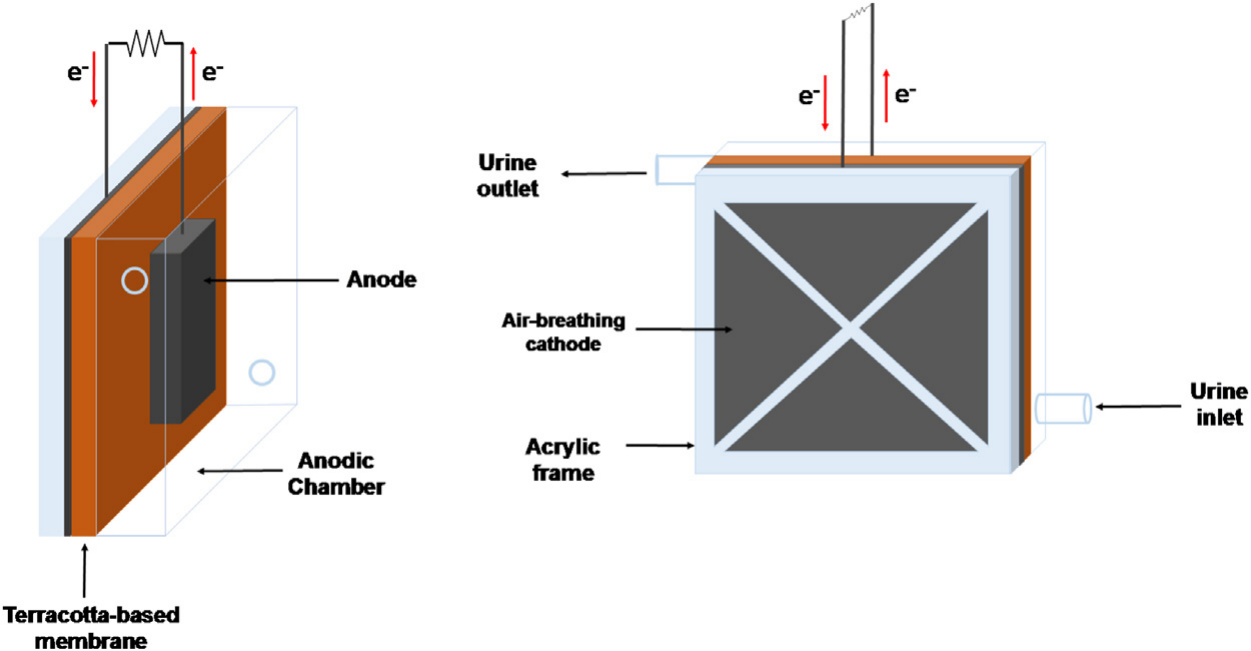
MFC set-up assessed.

Cubical ceramic-based MFC set-up was selected in order to analyse the effect of three operating parameters such as anode area, membrane thickness and external resistance on the power performance. The anode consists of a piece of carbon veil (30 g m^−2^, PRF composites, Dorset, UK) coated with activated carbon (AC. GBaldwin&Co., UK) and placed in an anode chamber with an empty volume of 12.5 mL. A chromium-nickel wire (0.4 mm, Scientific Wire Company) was used as the current collector. Regarding the cathode, it was made of a blend of activated carbon and polytetrafluoroethylene (80-20) pressed over a stainless steel mesh and exposed to air. Flat terracotta membranes were handmade by kilning square pieces of terracotta clay (3.5 × 3.5 cm^2^) for 3 min at 1070 °C. Fig. 2 shows the MFC set-up used in this work.

MFCs were initially inoculated with 1:1 ratio of sludge (Wessex Water Scientific Laboratory, Saltford, UK) and human urine in batch mode. After four cycles (one day each) in which the solution was completely replenished, MFCs were fed only with urine in continuous flow (0.1 mL min^−1^) during 360 h and the voltage was continuously monitored by an Agilent data logger (LXI 34972A data acquisition/Switch unit). Polarisation experiments were conducted as part of the characterisation work, however for the sake of clarity, these data are not shown. In order to optimise the performance and understand the behaviour of cubical ceramic-based MFCs, the effect of three different operating parameters on their energy harvesting was evaluated. In particular, three different anode areas (182.25 cm^2^, 100.25 cm^2^, 22.25 cm^2^), terracotta thicknesses (2.2 mm, 1.6 mm and 1.0 mm) and external loads (1,400 Ω, 710 Ω and 20 Ω) were assessed by performing 15-parameter combination in triplicate (45 tests).

### 2.2 Statistical analysis

The aim of RSM design is to maximise the response variable of a system by analysing which factor has the largest effect on this variable. The most important advantage of this methodology is that it considers both the effect of each individual factor and their interactions on the response-variable. So far, most of MFCs model reported in literature are based on systems fed with simple substrates such as acetate or glucose. However, in this work ceramic-MFCs are fed with real human urine. Due to the complexity of the system, RSM was selected as simple and fast empirical tool to maximise the MFC performance.

The effect model of a BBM design can be written as following:

(1) $$y=\beta_{0}+\sum_{i=1}^{k}\beta_{i}x_{i}+\sum_{i=1}^{k}\beta_{\mathrm{ii}}x_{i}^{2}+\sum_{j=2}^{k}\sum_{i=1}^{j-1}\beta_{\mathrm{ij}}x_{i}x_{j}+\varepsilon ,$$

where *β*_*i*_ and *β*_*ii*_ are the coefficient of the *i*th main effect and its quadratic effect, respectively, *β*_*ij*_ is the coefficient of the interaction between *i*th and *j*th factors, *β*_0_ is the independent coefficient and *ε* is the random error. The statistical analysis of the experimental design was performed by using the commercial Data Analysis software Statgraphics Centurion 18 © (version 18.1.06) and Minitab 18 © (version 18.1).

## 3 Results and discussion

A three-factor three level Box–Behnken design methodology was used for designing the optimisation process of cubical ceramic-based MFCs. A total number of 15 runs were performed in triplicate in a single base block, since all the tests were conducted under steady state conditions. Table 1 summarises the setting factor design for each experimental run. From this point onwards, in terms of the statistical analysis, the effect of the membrane thickness will be labelled as “A”, the effect of the external resistance as “B”, the effect of the anode area as “C” and the interaction between all of them as “AB”, “AC” and “BC”, respectively. For each parameter, -1 is considered as the minimum, 0 the medium and 1 the maximum value of the range selected.

**Table 1:** Design table of the setting value for each experimental run.

Run	A	B	C
1	−1	−1	0
2	1	−1	0
3	−1	1	0
4	1	1	0
5	−1	0	−1
6	1	0	−1
7	−1	0	1
8	1	0	1
9	0	−1	−1
10	0	1	−1
11	0	−1	1
12	0	1	1
13	0	0	0
14	0	0	0
15	0	0	0

The experimental results obtained by the 15-parameter combinations assessed in triplicate are depicted in Table 2. As can be observed, the maximum absolute power output in steady state (471.46 μW) is reached when MFCs work with an anode area of 182.25 cm^2^, a membrane thickness of 1 mm and an external load of 710 Ω.

**Table 2:** Experimental values of stationary power output by the cubical MFCs fed with urine under the operating conditions selected.

Run	A: thickness (mm)	B: external resistance (Ω)	C: anode area (cm^2^)	Experimental power (μW)	Standardised experimental power (μW cm^−2^)
1	1.0	1,400	102.25	247.39	2.42
2	2.2	1,400	102.25	249.63	2.44
3	1.0	20	102.25	29.05	0.28
4	2.2	20	102.25	18.07	0.18
5	1.0	710	22.25	62.94	2.83
6	2.2	710	22.25	3.640	1.38
7	1.0	710	182.25	471.46	2.59
8	2.2	710	182.25	433.77	2.38
9	1.6	1,400	22.25	146.47	6.58
10	1.6	20	22.25	0.19	0.01
11	1.6	1,400	182.25	270.72	1.49
12	1.6	20	182.25	84.96	0.47
13	1.6	710	102.25	422.52	4.14
14	1.6	710	102.25	382.85	3.74
15	1.6	710	102.25	405.20	3.96

It is also worth mentioning that from a scaling/normalisation perspective, it was found that the MFCs with the smallest anode surface area (22.25 cm^2^) outperformed those with the largest anode surface area (182.25 cm^2^), whereby the power density was 6.58 μW.cm^2^ (small anode) vs 1.49 μW cm^2^ (large anode) for a membrane thickness of 1.6 mm, both under an external loading of 1,400 Ω. This is also in agreement with the literature [26] and the reason why the normalised data have been included.

Eq. (1) was solved in the following forms: linear, quadratic (without interactions) and full quadratic (including interactions). All of them were analysed by ANOVA and regression coefficient (r^2^), residual sum of squares (RSS) and lack-of-fit (*p*-value) were determined (see Table 3).

**Table 3:** Analysis of variance (ANOVA) in power.

					
Analysis of variance (ANOVA)					
Source	DF	Adj SS	Adj MS	*F*-value	*p*-value
Model	9	376,191	41,799	4.32	0.061

Linear	3	207,440	69,147	7.14	0.029
A (mm)	1	776	776	0.08	0.788
B (Ω)	1	76,442	76,442	7.90	0.038
C (cm^2^)	1	130,221	130,221	13.45	0.014

Square	3	168,310	56,103	5.80	0.044
A (mm) × A (mm)	1	18,979	18,979	1.96	0.22
A (mm) × C (cm^2^)	1	141,571	141,571	14.63	0.012
B (Ω) × C (cm^2^)	1	24,904	24,904	2.57	0.170

2-Way interaction	3	441	147	0.02	0.997
A (mm) × B (Ω)	1	44	44	0.00	0.949
A (mm) × C (cm^2^)	1	7	7	0.00	0.979
B (mm) × C (cm^2^)	1	390	390	0.04	0.849

Error	5	48,393	9,679		
Lack-of-fit	3	47,602	15,867	40.12	0.024
Pure error	2	791	15,867		

Total	14	424,583	396		

The analysis of variance in power allows us to obtain a second order model, which maximises the power harvesting in ceramic-based MFCs, where the value of each variable is specified in their original units:

(2) $$\begin{array}{cc} P & =-668+618\times A+0.69\times B+4.14\times C-199\times A^{2}-0.000411\times B^{2}\\ & -0.01283\times C^{2}+0.008\times A\times B-0.03\times A\times C+0.000179\times B\times C \end{array}$$

The coefficient of determination of 0.886 implies that the model is able to express approximately 88.6% of the variability in the response. It should be noted that there is a lack-of-fit for the combination of the extreme values of B and C investigated, 20.25 cm^2^ and 20 Ω respectively. It might be due to the selection of too small an anode area combined with a very low external resistance having a negative effect on the biofilm growth. The development of a weak anode might also affect the reproducibility of the MFC behaviour under these extreme operating conditions, reducing the accuracy of the model in that region. These results are in line with those reported by Pasternak et al. [27]. Nevertheless, the regression coefficient is sufficiently high to consider that Eq. (2) describes fairly well the effect of the membrane thickness, the anode area and the external resistance on the power output by terracotta-based MFCs.

However, the variance analysis reports that only the main factors B and C, along with the main interaction BB have a significant effect with 95% confidence (*p* < 0.05). *p*-Values lower than 0.05 indicates a significant effect whereas *p*-values higher than 0.05 reports the contrary. In consideration of the results showed in Table 3, neither the main effect nor the interaction of the membrane thickness has a significant effect over the power output. This low influence might be due to the range of values selected. The range was selected according to a previous work focuses on fire fine clay-based MFCs [28]. This work reports that the MFC performance increases as the membrane thickness decreases, with maximum power recorded for a thickness of 2.5 mm. In that case, the membrane thicknesses ranged between 10 mm and 2.5 mm. Based on this previous work, the range of the membrane thickness selected was below 2.5 mm in order to analyse the behaviour of the system for thinner membranes [28].

**Fig. 3:**
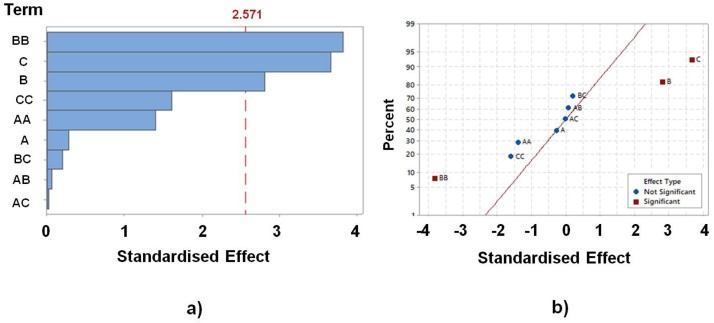
Standardised effects plots for power (*α* = 0.05): (a) Pareto chart and (b) normal plot.

The analysis of the standardised effects plot delivers the same conclusion than variance analysis. Fig. 3a shows the Pareto chart which depicts the standardised effects with *p* = 0.05. The bar length belongs to the absolute standardised value. Only the bars related to both factors B and C, as well as the interaction BB overcome the reference line (2.571), being the only effects statistically significant. The significant contribution of the BB quadratic effect reports the presence of a curvature over the response surface associated with the model.

Since Pareto chart depicts the absolute values, it does not allow us to determine whether the effects increase or reduce the value of the response variable. These results are achieved by the normal plot of standardised effects (see Fig. 3b). It allows us to display the magnitude as well as the direction of the effects. This chart shows the standardised effects to a distribution fit line for the case when all the effects are 0. The effects placed on the left side of the line have a negative influence on the output variable (BB) whereas the effects placed on the right side of the line have a positive influence (B and C). The furthest effects from the adjustment line show the most significant influence on the model.

**Fig. 4:**
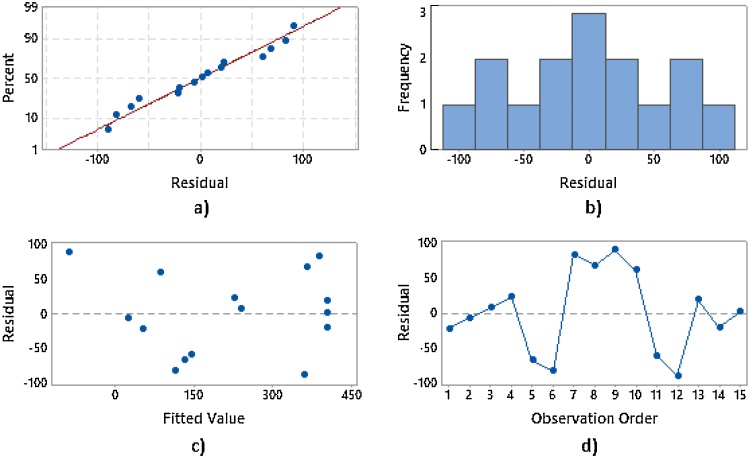
Residual plot for power: (a) normal probability plot, (b) histogram, (c) residual vs fitted value and (d) residual vs observation order.

On the other hand, the residual analysis, which shows the difference between the real value and the adjusted value, allows us to examine the goodness-of-fit in regression and ANOVA. Fig. 4 contains different residual plot for power. The normal probability plot approximates to a straight line, which demonstrates the normal distribution of the residues. The symmetry of the residual histogram also confirms the normal distribution of the results. The residual versus the fit plot, as well as the residual versus the order plot demonstrate the independence of the residual since they are randomly distributed on both sides of 0, without following any pattern. The presence of pattern on the residual distribution might indicate that the assumptions of the model are not met.

According to these results, the model equation might be simplified by removing those factors non-statistically significant:

(3) $$P=-143.7+0.693\times B+1.595\times C+0.000388\times B^{2}$$

**Fig. 5:**
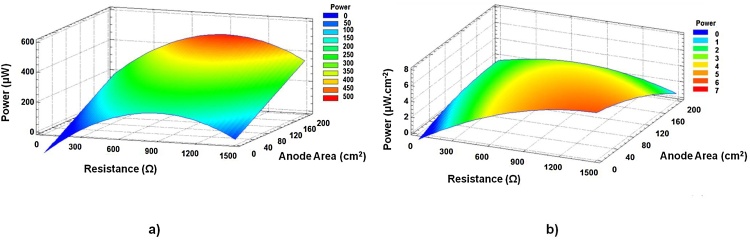
Estimated response surface for power (membrane thickness = 1.6 mm): (a) absolute power and (b) normalised power to anode area.

Fig. 5 plots the power surface response as a function of both anode area and external resistance for a specific membrane thickness of 1.6 mm. It should be noted that the effect of anode area on the power output is relatively higher than the external resistance. These results are in line with those reported in Fig. 3. As can be observed, anode area of ca. 160 cm^2^, as well as external loading around 900 Ω, would allow MFCs to reach the maximum absolute power output (see Fig. 5a). However, in terms of normalised anode, it is worth mentioning that the smallest surface area is more efficient than the largest one.

## 4 Conclusions

The aim of this work was to use a response surface analysis methodology in order to design a series of experiments for optimising the performance of cubical ceramic-based MFCs fed with urine. In this case, a Box–Behnken design was used for determining the influence of three operating parameters such as membrane thickness, external resistance and anode area on the MFC performance with a total number of 45 assays performed.

The three-factor three-level Box-Behnken designed allows us to determine a second order model for the system investigated with a regression coefficient (*r*^2^) of 0.886. The model shows that the theoretical maximum power output is 467.12 μW. Regarding the optimisation of the operating parameters for maximising the absolute power output, the resolution of the quadratic equation shows that the theoretical optimum membrane thickness, external loading and anode area are 1.55 mm, 895.59 Ω and 165.72 cm^2^, respectively. However, from a normalisation perspective, the smallest anode surface area gave the highest power density output, which would be the design parameter used in implementing a larger scale system. On the other hand, the variance analysis in power reports that anode area and external resistance, as well as the quadratic effect of the external resistance, have more influence on the performance of the MFC set-up studied than membrane thickness, within the selected range. The statistical based response surface methodology used in this work is a useful and simple tool for evaluating which operating factors have more influence on the MFC performance as well as optimising their values in a quadratic surface response. The optimum parameters identified by three-factor/three-level Box–Behnken can and will be used to inform the next line of experiments, which can go beyond the parameters tested herein. Finally, this model could also be used with other geometries based on the same materials to help identify the optimum surface area and load values and therefore save valuable design and set-up time for any practical application.
